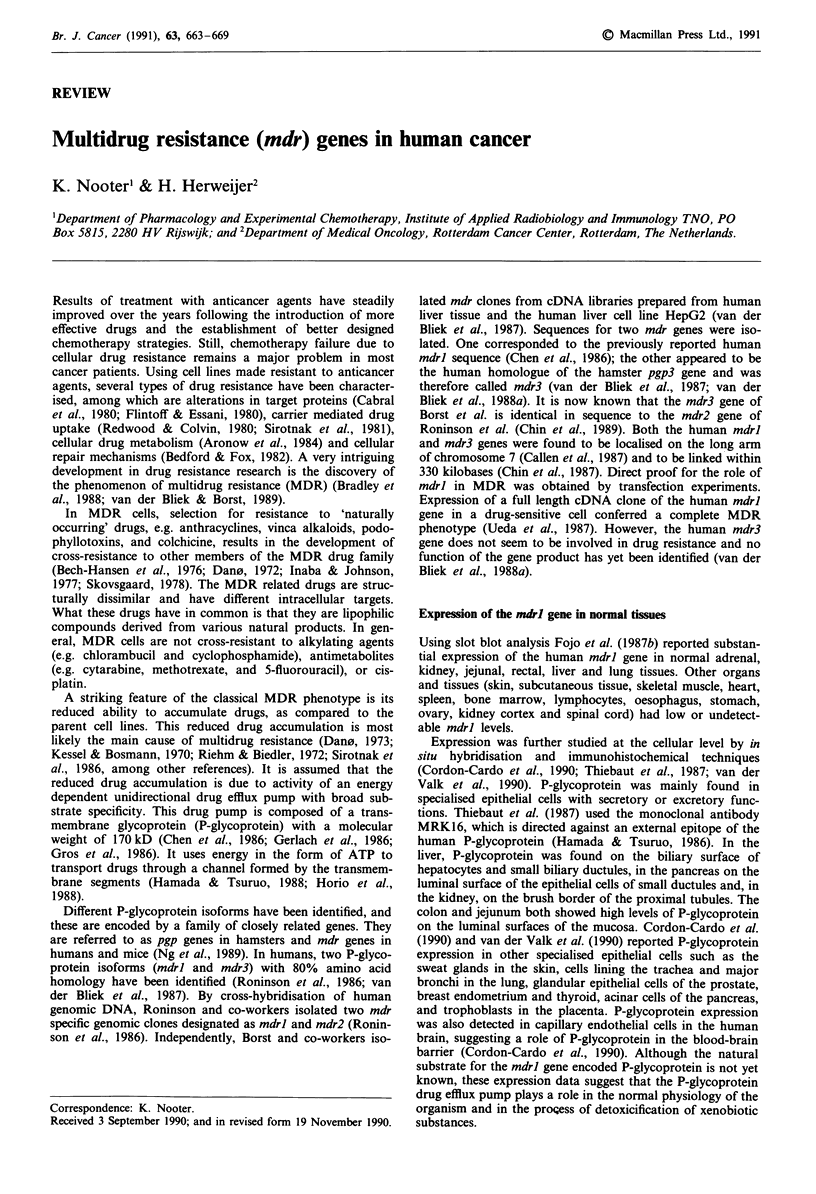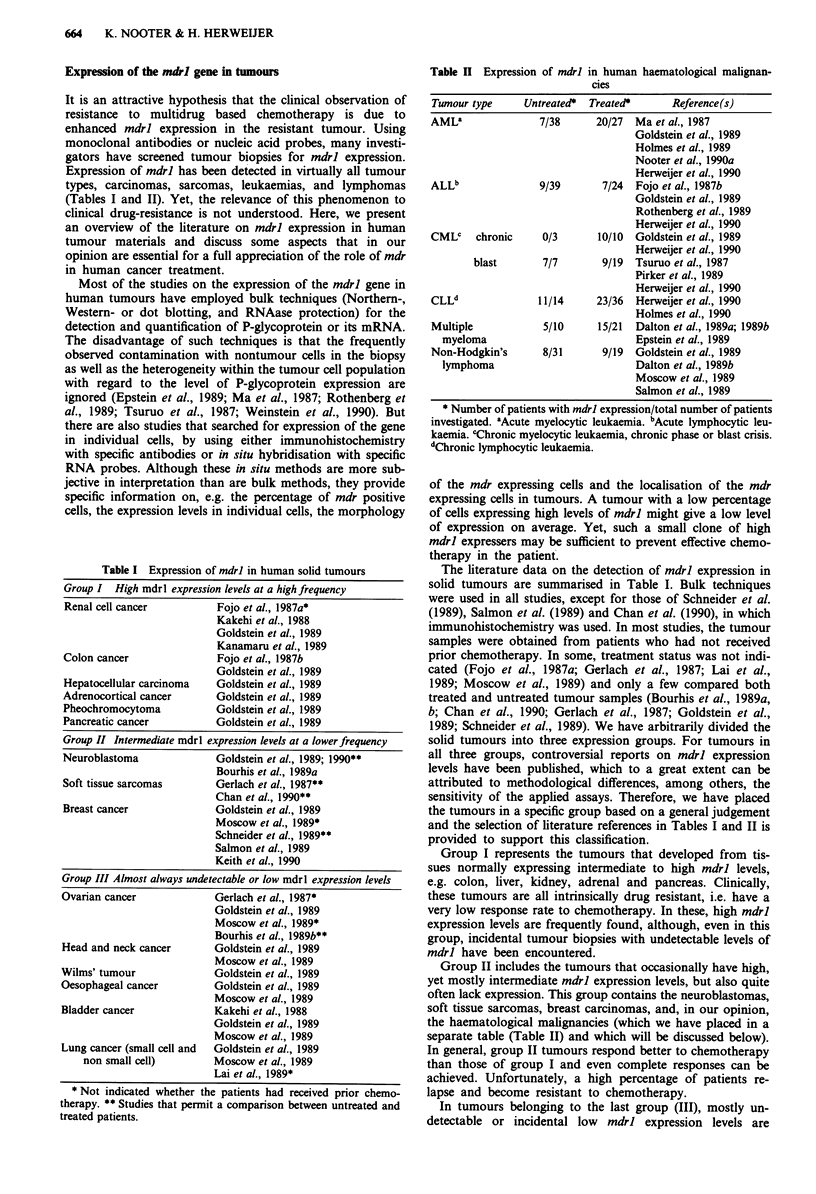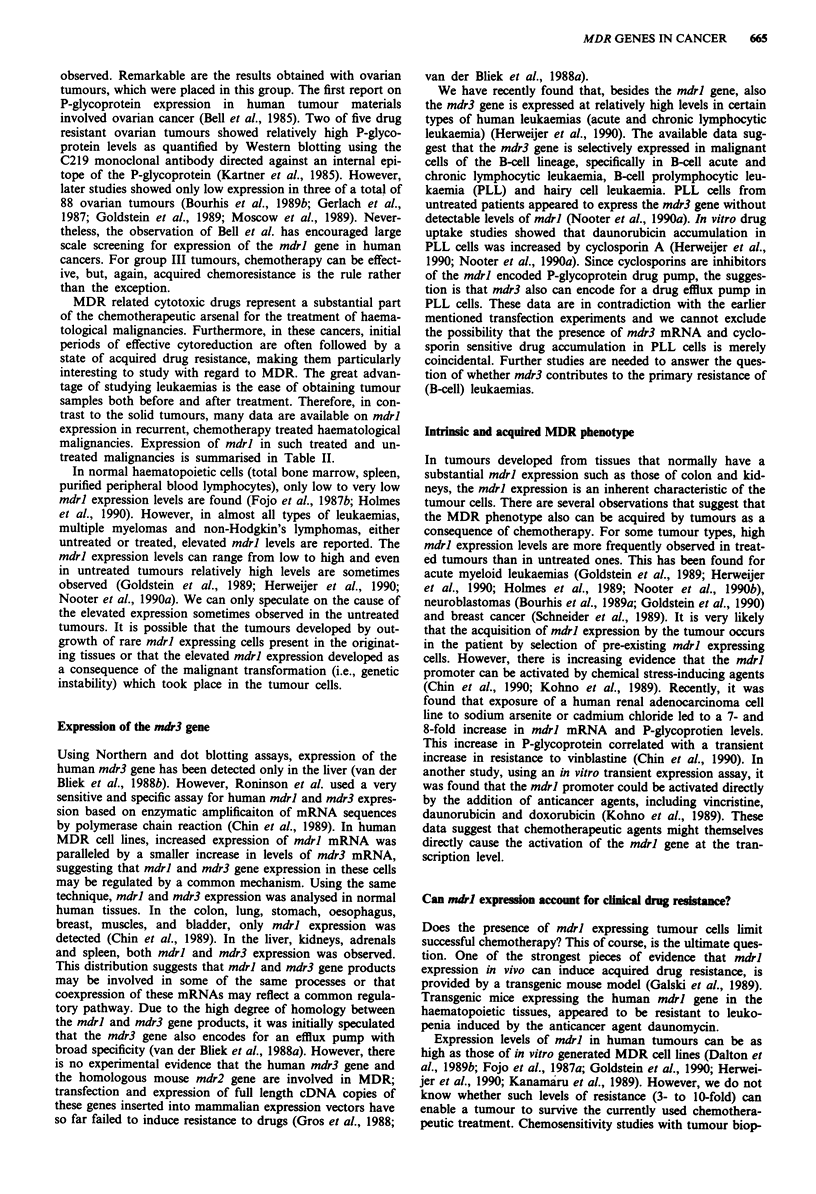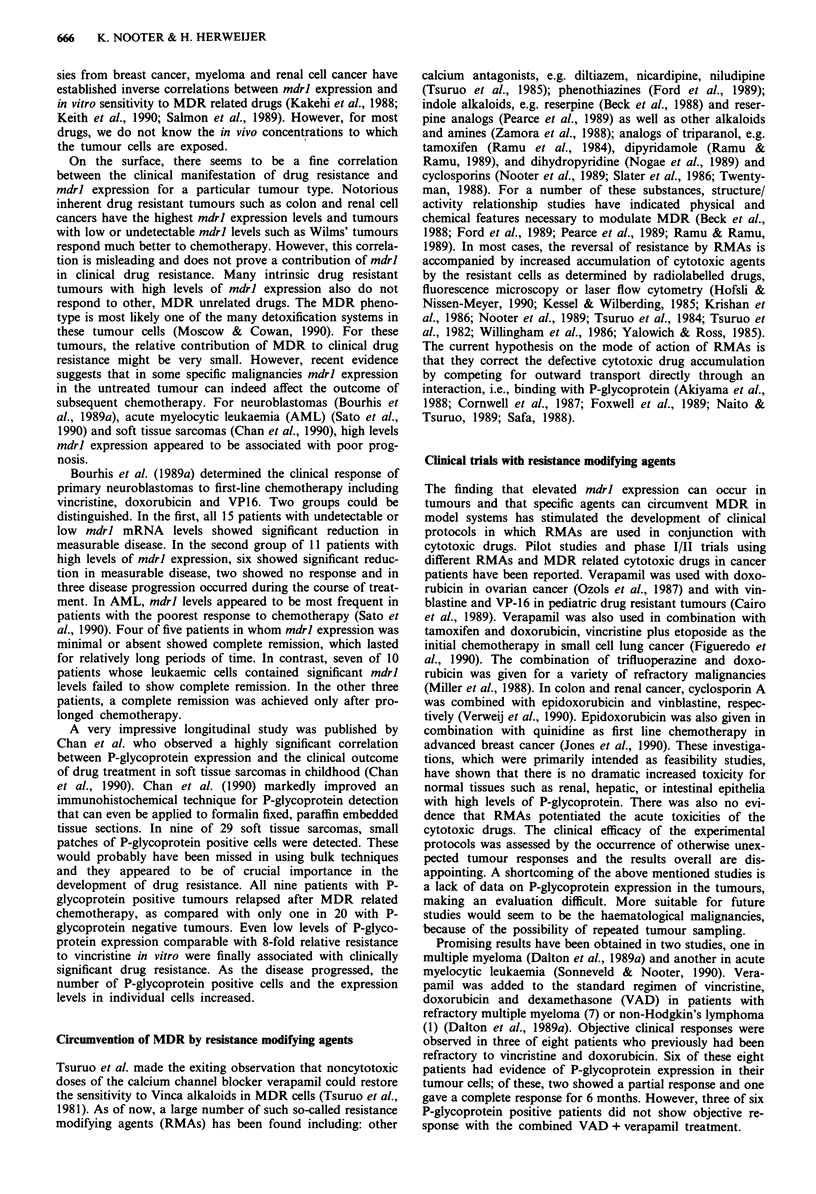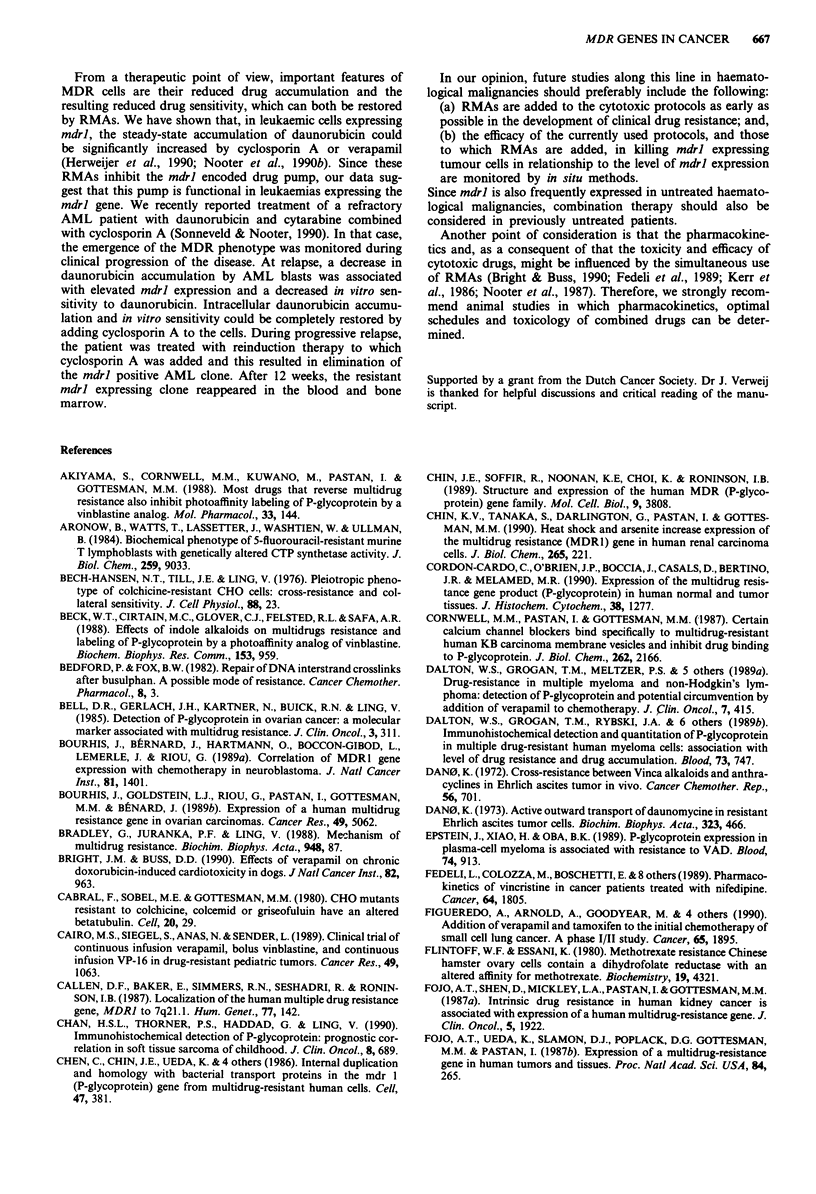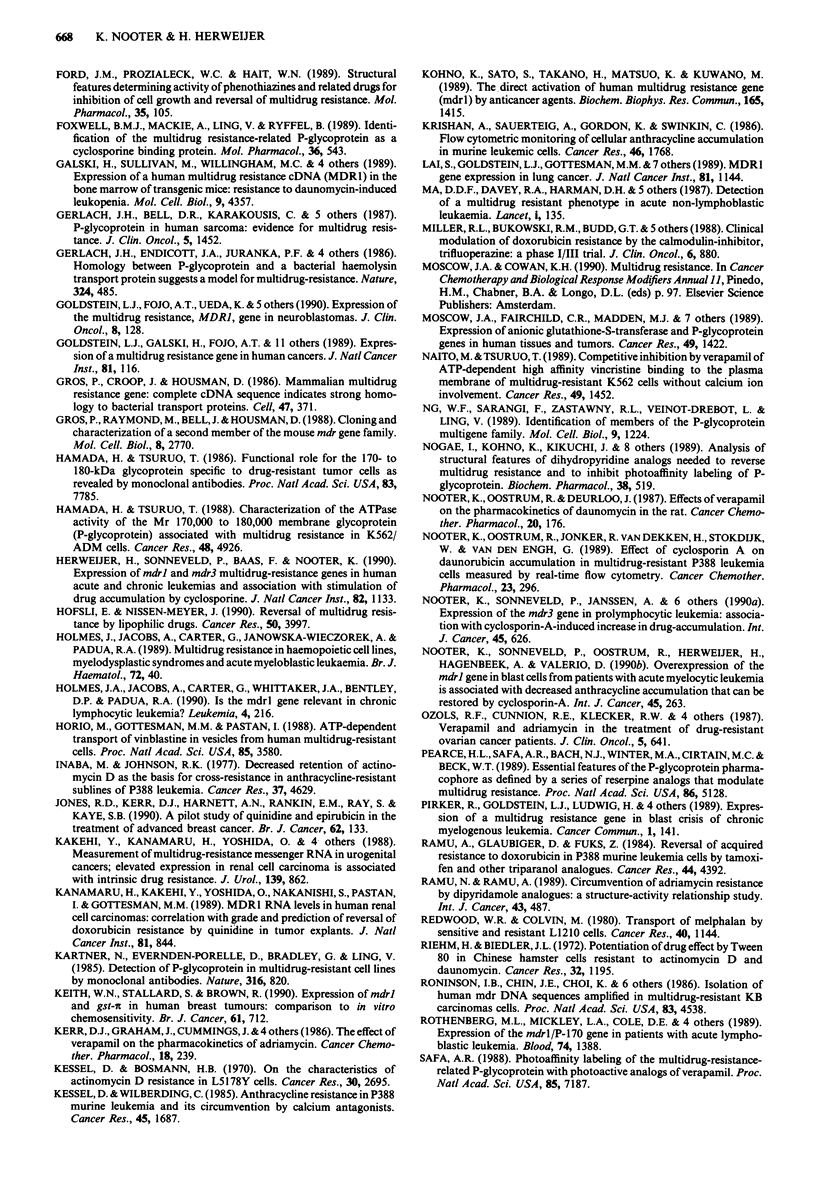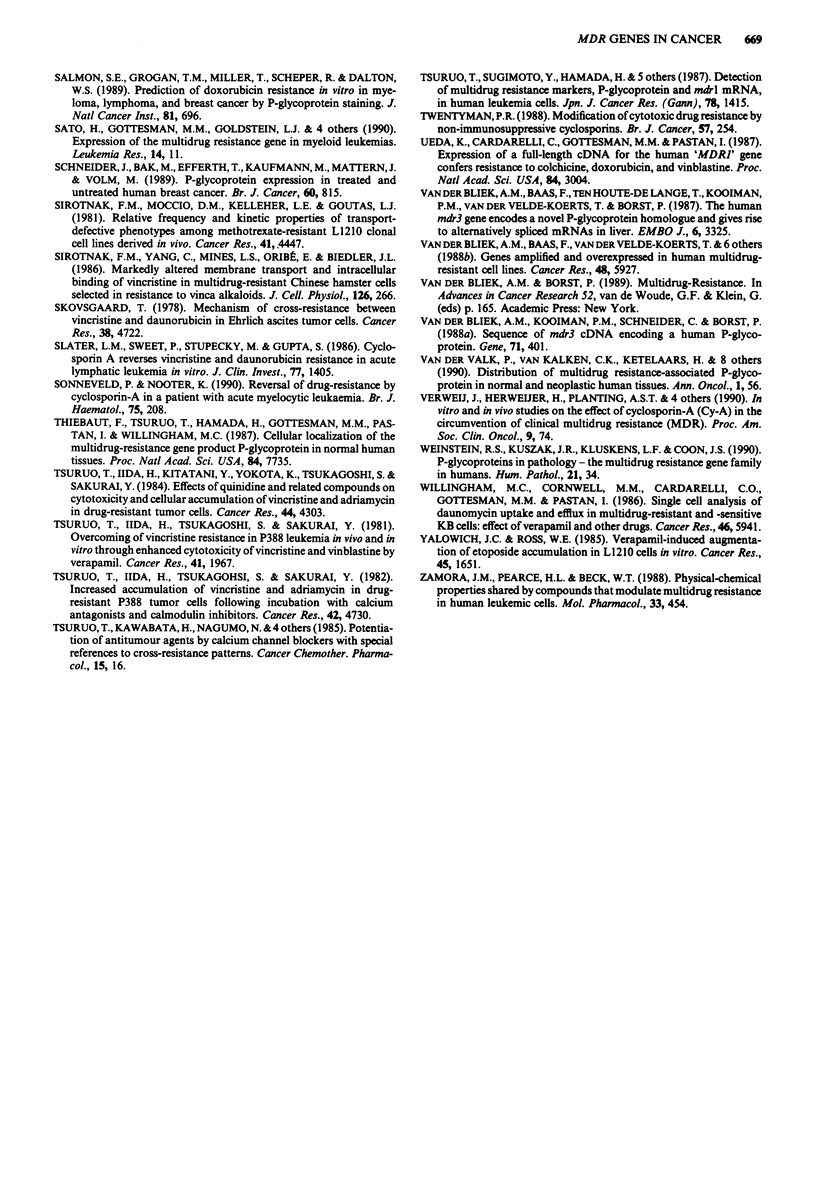# Multidrug resistance (mdr) genes in human cancer.

**DOI:** 10.1038/bjc.1991.152

**Published:** 1991-05

**Authors:** K. Nooter, H. Herweijer

**Affiliations:** Department of Pharmacology and Experimental Chemotherapy, Institute of Applied Radiobiology and Immunology TNO, Rijswijk, The Netherlands.


Br. J. Cancer (1991), 63, 663-669 © Macmillan Press Ltd., 1991

REVIEW

### Multidrug resistance (*mdr*) genes in human cancer

K. Nooter[1] & H. Herweijer[2]

[1]Department of Pharmacology and Experimental Chemotherapy, Institute of Applied Radiobiology and Immunology TNO, PO Box 5815, 2280 HV Rijswijk; and [2]Department of Medical Oncology, Rotterdam Cancer Center, Rotterdam, The Netherlands.

Results of treatment with anticancer agents have steadily improved over the years following the introduction of more effective drugs and the establishment of better designed chemotherapy strategies. Still, chemotherapy failure due to cellular drug resistance remains a major problem in most cancer patients. Using cell lines made resistant to anticancer agents, several types of drug resistance have been characterised, among which are alterations in target proteins (Cabral *et al.*, 1980; Flintoff & Essani, 1980), carrier mediated drug uptake (Redwood & Colvin, 1980; Sirotnak *et al.*, 1981), cellular drug metabolism (Aronow *et al.*, 1984) and cellular repair mechanisms (Bedford & Fox, 1982). A very intriguing development in drug resistance research is the discovery of the phenomenon of multidrug resistance (MDR) (Bradley *et al.*, 1988; van der Bliek & Borst, 1989).

In MDR cells, selection for resistance to 'naturally occurring' drugs, e.g. anthracyclines, vinca alkaloids, podophyllotoxins, and colchicine, results in the development of cross-resistance to other members of the MDR drug family (Bech-Hansen *et al.*, 1976; Danø, 1972; Inaba & Johnson, 1977; Skovsgaard, 1978). The MDR related drugs are structurally dissimilar and have different intracellular targets. What these drugs have in common is that they are lipophilic compounds derived from various natural products. In general, MDR cells are not cross-resistant to alkylating agents (e.g. chlorambucil and cyclophosphamide), antimetabolites (e.g. cytarabine, methotrexate, and 5-fluorouracil), or cisplatin.

A striking feature of the classical MDR phenotype is its reduced ability to accumulate drugs, as compared to the parent cell lines. This reduced drug accumulation is most likely the main cause of multidrug resistance (Danø, 1973; Kessel & Bosmann, 1970; Riehm & Biedler, 1972; Sirotnak *et al.*, 1986, among other references). It is assumed that the reduced drug accumulation is due to activity of an energy dependent unidirectional drug efflux pump with broad substrate specificity. This drug pump is composed of a transmembrane glycoprotein (P-glycoprotein) with a molecular weight of 170 kD (Chen *et al.*, 1986; Gerlach *et al.*, 1986; Gros *et al.*, 1986). It uses energy in the form of ATP to transport drugs through a channel formed by the transmembrane segments (Hamada & Tsuruo, 1988; Horio *et al.*, 1988).

Different P-glycoprotein isoforms have been identified, and these are encoded by a family of closely related genes. They are referred to as *pgp* genes in hamsters and *mdr* genes in humans and mice (Ng *et al.*, 1989). In humans, two P-glycoprotein isoforms (*mdr1* and *mdr3*) with 80% amino acid homology have been identified (Roninson *et al.*, 1986; van der Bliek *et al.*, 1987). By cross-hybridisation of human genomic DNA, Roninson and co-workers isolated two *mdr* specific genomic clones designated as *mdr1* and *mdr2* (Roninson *et al.*, 1986). Independently, Borst and co-workers isolated *mdr* clones from cDNA libraries prepared from human liver tissue and the human liver cell line HepG2 (van der Bliek *et al.*, 1987). Sequences for two *mdr* genes were isolated. One corresponded to the previously reported human *mdr1* sequence (Chen *et al.*, 1986); the other appeared to be the human homologue of the hamster *pgp3* gene and was therefore called *mdr3* (van der Bliek *et al.*, 1987; van der Bliek *et al.*, 1988*a*). It is now known that the *mdr3* gene of Borst *et al.* is identical in sequence to the *mdr2* gene of Roninson *et al.* (Chin *et al.*, 1989). Both the human *mdr1* and *mdr3* genes were found to be localised on the long arm of chromosome 7 (Callen *et al.*, 1987) and to be linked within 330 kilobases (Chin *et al.*, 1987). Direct proof for the role of *mdr1* in MDR was obtained by transfection experiments. Expression of a full length cDNA clone of the human *mdr1* gene in a drug-sensitive cell conferred a complete MDR phenotype (Ueda *et al.*, 1987). However, the human *mdr3* gene does not seem to be involved in drug resistance and no function of the gene product has yet been identified (van der Bliek *et al.*, 1988*a*).

### Expression of the *mdr1* gene in normal tissues

Using slot blot analysis Fojo *et al.* (1987*b*) reported substantial expression of the human *mdr1* gene in normal adrenal, kidney, jejunal, rectal, liver and lung tissues. Other organs and tissues (skin, subcutaneous tissue, skeletal muscle, heart, spleen, bone marrow, lymphocytes, oesophagus, stomach, ovary, kidney cortex and spinal cord) had low or undetectable *mdr1* levels.

Expression was further studied at the cellular level by *in situ* hybridisation and immunohistochemical techniques (Cordon-Cardo *et al.*, 1990; Thiebaut *et al.*, 1987; van der Valk *et al.*, 1990). P-glycoprotein was mainly found in specialised epithelial cells with secretory or excretory functions. Thiebaut *et al.* (1987) used the monoclonal antibody MRK16, which is directed against an external epitope of the human P-glycoprotein (Hamada & Tsuruo, 1986). In the liver, P-glycoprotein was found on the biliary surface of hepatocytes and small biliary ductules, in the pancreas on the luminal surface of the epithelial cells of small ductules and, in the kidney, on the brush border of the proximal tubules. The colon and jejunum both showed high levels of P-glycoprotein on the luminal surfaces of the mucosa. Cordon-Cardo *et al.* (1990) and van der Valk *et al.* (1990) reported P-glycoprotein expression in other specialised epithelial cells such as the sweat glands in the skin, cells lining the trachea and major bronchi in the lung, glandular epithelial cells of the prostate, breast endometrium and thyroid, acinar cells of the pancreas, and trophoblasts in the placenta. P-glycoprotein expression was also detected in capillary endothelial cells in the human brain, suggesting a role of P-glycoprotein in the blood-brain barrier (Cordon-Cardo *et al.*, 1990). Although the natural substrate for the *mdr1* gene encoded P-glycoprotein is not yet known, these expression data suggest that the P-glycoprotein drug efflux pump plays a role in the normal physiology of the organism and in the process of detoxicification of xenobiotic substances.

Correspondence: K. Nooter.
Received 3 September 1990; and in revised form 19 November 1990.

### Expression of the *mdr1* gene in tumours

It is an attractive hypothesis that the clinical observation of resistance to multidrug based chemotherapy is due to enhanced *mdr1* expression in the resistant tumour. Using monoclonal antibodies or nucleic acid probes, many investigators have screened tumour biopsies for *mdr1* expression. Expression of *mdr1* has been detected in virtually all tumour types, carcinomas, sarcomas, leukaemias, and lymphomas (Tables I and II). Yet, the relevance of this phenomenon to clinical drug-resistance is not understood. Here, we present an overview of the literature on *mdr1* expression in human tumour materials and discuss some aspects that in our opinion are essential for a full appreciation of the role of *mdr* in human cancer treatment.

Most of the studies on the expression of the *mdr1* gene in human tumours have employed bulk techniques (Northern-, Western- or dot blotting, and RNAase protection) for the detection and quantification of P-glycoprotein or its mRNA. The disadvantage of such techniques is that the frequently observed contamination with nontumour cells in the biopsy as well as the heterogeneity within the tumour cell population with regard to the level of P-glycoprotein expression are ignored (Epstein *et al.*, 1989; Ma *et al.*, 1987; Rothenberg *et al.*, 1989; Tsuruo *et al.*, 1987; Weinstein *et al.*, 1990). But there are also studies that searched for expression of the gene in individual cells, by using either immunohistochemistry with specific antibodies or *in situ* hybridisation with specific RNA probes. Although these *in situ* methods are more subjective in interpretation than are bulk methods, they provide specific information on, e.g. the percentage of *mdr* positive cells, the expression levels in individual cells, the morphology of the *mdr* expressing cells and the localisation of the *mdr* expressing cells in tumours. A tumour with a low percentage of cells expressing high levels of *mdr1* might give a low level of expression on average. Yet, such a small clone of high *mdr1* expressers may be sufficient to prevent effective chemotherapy in the patient.

**Table I** Expression of *mdr1* in human solid tumours

| | |
|---|---|
| *Group I High* mdr1 *expression levels at a high frequency* | |
| Renal cell cancer | Fojo *et al.*, 1987*a** |
| | Kakehi *et al.*, 1988 |
| | Goldstein *et al.*, 1989 |
| | Kanamaru *et al.*, 1989 |
| Colon cancer | Fojo *et al.*, 1987*b* |
| | Goldstein *et al.*, 1989 |
| Hepatocellular carcinoma | Goldstein *et al.*, 1989 |
| Adrenocortical cancer | Goldstein *et al.*, 1989 |
| Pheochromocytoma | Goldstein *et al.*, 1989 |
| Pancreatic cancer | Goldstein *et al.*, 1989 |
| *Group II Intermediate* mdr1 *expression levels at a lower frequency* | |
| Neuroblastoma | Goldstein *et al.*, 1989; 1990** |
| | Bourhis *et al.*, 1989*a* |
| Soft tissue sarcomas | Gerlach *et al.*, 1987** |
| | Chan *et al.*, 1990** |
| Breast cancer | Goldstein *et al.*, 1989 |
| | Moscow *et al.*, 1989* |
| | Schneider *et al.*, 1989** |
| | Salmon *et al.*, 1989 |
| | Keith *et al.*, 1990 |
| *Group III Almost always undetectable or low* mdr1 *expression levels* | |
| Ovarian cancer | Gerlach *et al.*, 1987* |
| | Goldstein *et al.*, 1989 |
| | Moscow *et al.*, 1989* |
| | Bourhis *et al.*, 1989*b*** |
| Head and neck cancer | Goldstein *et al.*, 1989 |
| | Moscow *et al.*, 1989 |
| Wilms' tumour | Goldstein *et al.*, 1989 |
| Oesophageal cancer | Goldstein *et al.*, 1989 |
| | Moscow *et al.*, 1989 |
| Bladder cancer | Kakehi *et al.*, 1988 |
| | Goldstein *et al.*, 1989 |
| | Moscow *et al.*, 1989 |
| Lung cancer (small cell and non small cell) | Goldstein *et al.*, 1989 |
| | Moscow *et al.*, 1989 |
| | Lai *et al.*, 1989* |

* Not indicated whether the patients had received prior chemotherapy. ** Studies that permit a comparison between untreated and treated patients.

**Table II** Expression of *mdr1* in human haematological malignancies

| *Tumour type* | | *Untreated** | *Treated** | *Reference(s)* |
|---|---|---|---|---|
| AML[a] | | 7/38 | 20/27 | Ma *et al.*, 1987 |
| | | | | Goldstein *et al.*, 1989 |
| | | | | Holmes *et al.*, 1989 |
| | | | | Nooter *et al.*, 1990*a* |
| | | | | Herweijer *et al.*, 1990 |
| ALL[b] | | 9/39 | 7/24 | Fojo *et al.*, 1987*b* |
| | | | | Goldstein *et al.*, 1989 |
| | | | | Rothenberg *et al.*, 1989 |
| | | | | Herweijer *et al.*, 1990 |
| CML[c] | chronic | 0/3 | 10/10 | Goldstein *et al.*, 1989 |
| | | | | Herweijer *et al.*, 1990 |
| | blast | 7/7 | 9/19 | Tsuruo *et al.*, 1987 |
| | | | | Pirker *et al.*, 1989 |
| | | | | Herweijer *et al.*, 1990 |
| CLL[d] | | 11/14 | 23/36 | Herweijer *et al.*, 1990 |
| | | | | Holmes *et al.*, 1990 |
| Multiple myeloma | | 5/10 | 15/21 | Dalton *et al.*, 1989*a*; 1989*b* |
| | | | | Epstein *et al.*, 1989 |
| Non-Hodgkin's lymphoma | | 8/31 | 9/19 | Goldstein *et al.*, 1989 |
| | | | | Dalton *et al.*, 1989*b* |
| | | | | Moscow *et al.*, 1989 |
| | | | | Salmon *et al.*, 1989 |

* Number of patients with *mdr1* expression/total number of patients investigated. [a]Acute myelocytic leukaemia. [b]Acute lymphocytic leukaemia. [c]Chronic myelocytic leukaemia, chronic phase or blast crisis. [d]Chronic lymphocytic leukaemia.

The literature data on the detection of *mdr1* expression in solid tumours are summarised in Table I. Bulk techniques were used in all studies, except for those of Schneider *et al.* (1989), Salmon *et al.* (1989) and Chan *et al.* (1990), in which immunohistochemistry was used. In most studies, the tumour samples were obtained from patients who had not received prior chemotherapy. In some, treatment status was not indicated (Fojo *et al.*, 1987*a*; Gerlach *et al.*, 1987; Lai *et al.*, 1989; Moscow *et al.*, 1989) and only a few compared both treated and untreated tumour samples (Bourhis *et al.*, 1989*a*, *b*; Chan *et al.*, 1990; Gerlach *et al.*, 1987; Goldstein *et al.*, 1989; Schneider *et al.*, 1989). We have arbitrarily divided the solid tumours into three expression groups. For tumours in all three groups, controversial reports on *mdr1* expression levels have been published, which to a great extent can be attributed to methodological differences, among others, the sensitivity of the applied assays. Therefore, we have placed the tumours in a specific group based on a general judgement and the selection of literature references in Tables I and II is provided to support this classification.

Group I represents the tumours that developed from tissues normally expressing intermediate to high *mdr1* levels, e.g. colon, liver, kidney, adrenal and pancreas. Clinically, these tumours are all intrinsically drug resistant, i.e. have a very low response rate to chemotherapy. In these, high *mdr1* expression levels are frequently found, although, even in this group, incidental tumour biopsies with undetectable levels of *mdr1* have been encountered.

Group II includes the tumours that occasionally have high, yet mostly intermediate *mdr1* expression levels, but also quite often lack expression. This group contains the neuroblastomas, soft tissue sarcomas, breast carcinomas, and, in our opinion, the haematological malignancies (which we have placed in a separate table (Table II) and which will be discussed below). In general, group II tumours respond better to chemotherapy than those of group I and even complete responses can be achieved. Unfortunately, a high percentage of patients relapse and become resistant to chemotherapy.

In tumours belonging to the last group (III), mostly undetectable or incidental low *mdr1* expression levels are observed. Remarkable are the results obtained with ovarian tumours, which were placed in this group. The first report on P-glycoprotein expression in human tumour materials involved ovarian cancer (Bell *et al.*, 1985). Two of five drug resistant ovarian tumours showed relatively high P-glycoprotein levels as quantified by Western blotting using the C219 monoclonal antibody directed against an internal epitope of the P-glycoprotein (Kartner *et al.*, 1985). However, later studies showed only low expression in three of a total of 88 ovarian tumours (Bourhis *et al.*, 1989*b*; Gerlach *et al.*, 1987; Goldstein *et al.*, 1989; Moscow *et al.*, 1989). Nevertheless, the observation of Bell *et al.* has encouraged large scale screening for expression of the *mdr1* gene in human cancers. For group III tumours, chemotherapy can be effective, but, again, acquired chemoresistance is the rule rather than the exception.

MDR related cytotoxic drugs represent a substantial part of the chemotherapeutic arsenal for the treatment of haematological malignancies. Furthermore, in these cancers, initial periods of effective cytoreduction are often followed by a state of acquired drug resistance, making them particularly interesting to study with regard to MDR. The great advantage of studying leukaemias is the ease of obtaining tumour samples both before and after treatment. Therefore, in contrast to the solid tumours, many data are available on *mdr1* expression in recurrent, chemotherapy treated haematological malignancies. Expression of *mdr1* in such treated and untreated malignancies is summarised in Table II.

In normal haematopoietic cells (total bone marrow, spleen, purified peripheral blood lymphocytes), only low to very low *mdr1* expression levels are found (Fojo *et al.*, 1987*b*; Holmes *et al.*, 1990). However, in almost all types of leukaemias, multiple myelomas and non-Hodgkin's lymphomas, either untreated or treated, elevated *mdr1* levels are reported. The *mdr1* expression levels can range from low to high and even in untreated tumours relatively high levels are sometimes observed (Goldstein *et al.*, 1989; Herweijer *et al.*, 1990; Nooter *et al.*, 1990*a*). We can only speculate on the cause of the elevated expression sometimes observed in the untreated tumours. It is possible that the tumours developed by outgrowth of rare *mdr1* expressing cells present in the originating tissues or that the elevated *mdr1* expression developed as a consequence of the malignant transformation (i.e., genetic instability) which took place in the tumour cells.

### Expression of the *mdr3* gene

Using Northern and dot blotting assays, expression of the human *mdr3* gene has been detected only in the liver (van der Bliek *et al.*, 1988*b*). However, Roninson *et al.* used a very sensitive and specific assay for human *mdr1* and *mdr3* expression based on enzymatic amplificaiton of mRNA sequences by polymerase chain reaction (Chin *et al.*, 1989). In human MDR cell lines, increased expression of *mdr1* mRNA was paralleled by a smaller increase in levels of *mdr3* mRNA, suggesting that *mdr1* and *mdr3* gene expression in these cells may be regulated by a common mechanism. Using the same technique, *mdr1* and *mdr3* expression was analysed in normal human tissues. In the colon, lung, stomach, oesophagus, breast, muscles, and bladder, only *mdr1* expression was detected (Chin *et al.*, 1989). In the liver, kidneys, adrenals and spleen, both *mdr1* and *mdr3* expression was observed. This distribution suggests that *mdr1* and *mdr3* gene products may be involved in some of the same processes or that coexpression of these mRNAs may reflect a common regulatory pathway. Due to the high degree of homology between the *mdr1* and *mdr3* gene products, it was initially speculated that the *mdr3* gene also encodes for an efflux pump with broad specificity (van der Bliek *et al.*, 1988*a*). However, there is no experimental evidence that the human *mdr3* gene and the homologous mouse *mdr2* gene are involved in MDR; transfection and expression of full length cDNA copies of these genes inserted into mammalian expression vectors have so far failed to induce resistance to drugs (Gros *et al.*, 1988; van der Bliek *et al.*, 1988*a*).

We have recently found that, besides the *mdr1* gene, also the *mdr3* gene is expressed at relatively high levels in certain types of human leukaemias (acute and chronic lymphocytic leukaemia) (Herweijer *et al.*, 1990). The available data suggest that the *mdr3* gene is selectively expressed in malignant cells of the B-cell lineage, specifically in B-cell acute and chronic lymphocytic leukaemia, B-cell prolymphocytic leukaemia (PLL) and hairy cell leukaemia. PLL cells from untreated patients appeared to express the *mdr3* gene without detectable levels of *mdr1* (Nooter *et al.*, 1990*a*). *In vitro* drug uptake studies showed that daunorubicin accumulation in PLL cells was increased by cyclosporin A (Herweijer *et al.*, 1990; Nooter *et al.*, 1990*a*). Since cyclosporins are inhibitors of the *mdr1* encoded P-glycoprotein drug pump, the suggestion is that *mdr3* also can encode for a drug efflux pump in PLL cells. These data are in contradiction with the earlier mentioned transfection experiments and we cannot exclude the possibility that the presence of *mdr3* mRNA and cyclosporin sensitive drug accumulation in PLL cells is merely coincidental. Further studies are needed to answer the question of whether *mdr3* contributes to the primary resistance of (B-cell) leukaemias.

### Intrinsic and acquired MDR phenotype

In tumours developed from tissues that normally have a substantial *mdr1* expression such as those of colon and kidneys, the *mdr1* expression is an inherent characteristic of the tumour cells. There are several observations that suggest that the MDR phenotype also can be acquired by tumours as a consequence of chemotherapy. For some tumour types, high *mdr1* expression levels are more frequently observed in treated tumours than in untreated ones. This has been found for acute myeloid leukaemias (Goldstein *et al.*, 1989; Herweijer *et al.*, 1990; Holmes *et al.*, 1989; Nooter *et al.*, 1990*b*), neuroblastomas (Bourhis *et al.*, 1989*a*; Goldstein *et al.*, 1990) and breast cancer (Schneider *et al.*, 1989). It is very likely that the acquisition of *mdr1* expression by the tumour occurs in the patient by selection of pre-existing *mdr1* expressing cells. However, there is increasing evidence that the *mdr1* promoter can be activated by chemical stress-inducing agents (Chin *et al.*, 1990; Kohno *et al.*, 1989). Recently, it was found that exposure of a human renal adenocarcinoma cell line to sodium arsenite or cadmium chloride led to a 7- and 8-fold increase in *mdr1* mRNA and P-glycoprotien levels. This increase in P-glycoprotein correlated with a transient increase in resistance to vinblastine (Chin *et al.*, 1990). In another study, using an *in vitro* transient expression assay, it was found that the *mdr1* promoter could be activated directly by the addition of anticancer agents, including vincristine, daunorubicin and doxorubicin (Kohno *et al.*, 1989). These data suggest that chemotherapeutic agents might themselves directly cause the activation of the *mdr1* gene at the transcription level.

### Can *mdr1* expression account for clinical drug resistance?

Does the presence of *mdr1* expressing tumour cells limit successful chemotherapy? This of course, is the ultimate question. One of the strongest pieces of evidence that *mdr1* expression *in vivo* can induce acquired drug resistance, is provided by a transgenic mouse model (Galski *et al.*, 1989). Transgenic mice expressing the human *mdr1* gene in the haematopoietic tissues, appeared to be resistant to leukopenia induced by the anticancer agent daunomycin.

Expression levels of *mdr1* in human tumours can be as high as those of *in vitro* generated MDR cell lines (Dalton *et al.*, 1989*b*; Fojo *et al.*, 1987*a*; Goldstein *et al.*, 1990; Herweijer *et al.*, 1990; Kanamaru *et al.*, 1989). However, we do not know whether such levels of resistance (3- to 10-fold) can enable a tumour to survive the currently used chemotherapeutic treatment. Chemosensitivity studies with tumour biopsies from breast cancer, myeloma and renal cell cancer have established inverse correlations between *mdr1* expression and *in vitro* sensitivity to MDR related drugs (Kakehi *et al.*, 1988; Keith *et al.*, 1990; Salmon *et al.*, 1989). However, for most drugs, we do not know the *in vivo* concentrations to which the tumour cells are exposed.

On the surface, there seems to be a fine correlation between the clinical manifestation of drug resistance and *mdr1* expression for a particular tumour type. Notorious inherent drug resistant tumours such as colon and renal cell cancers have the highest *mdr1* expression levels and tumours with low or undetectable *mdr1* levels such as Wilms' tumours respond much better to chemotherapy. However, this correlation is misleading and does not prove a contribution of *mdr1* in clinical drug resistance. Many intrinsic drug resistant tumours with high levels of *mdr1* expression also do not respond to other, MDR unrelated drugs. The MDR phenotype is most likely one of the many detoxification systems in these tumour cells (Moscow & Cowan, 1990). For these tumours, the relative contribution of MDR to clinical drug resistance might be very small. However, recent evidence suggests that in some specific malignancies *mdr1* expression in the untreated tumour can indeed affect the outcome of subsequent chemotherapy. For neuroblastomas (Bourhis *et al.*, 1989*a*), acute myelocytic leukaemia (AML) (Sato *et al.*, 1990) and soft tissue sarcomas (Chan *et al.*, 1990), high levels *mdr1* expression appeared to be associated with poor prognosis.

Bourhis *et al.* (1989*a*) determined the clinical response of primary neuroblastomas to first-line chemotherapy including vincristine, doxorubicin and VP16. Two groups could be distinguished. In the first, all 15 patients with undetectable or low *mdr1* mRNA levels showed significant reduction in measurable disease. In the second group of 11 patients with high levels of *mdr1* expression, six showed significant reduction in measurable disease, two showed no response and in three disease progression occurred during the course of treatment. In AML, *mdr1* levels appeared to be most frequent in patients with the poorest response to chemotherapy (Sato *et al.*, 1990). Four of five patients in whom *mdr1* expression was minimal or absent showed complete remission, which lasted for relatively long periods of time. In contrast, seven of 10 patients whose leukaemic cells contained significant *mdr1* levels failed to show complete remission. In the other three patients, a complete remission was achieved only after prolonged chemotherapy.

A very impressive longitudinal study was published by Chan *et al.* who observed a highly significant correlation between P-glycoprotein expression and the clinical outcome of drug treatment in soft tissue sarcomas in childhood (Chan *et al.*, 1990). Chan *et al.* (1990) markedly improved an immunohistochemical technique for P-glycoprotein detection that can even be applied to formalin fixed, paraffin embedded tissue sections. In nine of 29 soft tissue sarcomas, small patches of P-glycoprotein positive cells were detected. These would probably have been missed in using bulk techniques and they appeared to be of crucial importance in the development of drug resistance. All nine patients with P-glycoprotein positive tumours relapsed after MDR related chemotherapy, as compared with only one in 20 with P-glycoprotein negative tumours. Even low levels of P-glycoprotein expression comparable with 8-fold relative resistance to vincristine *in vitro* were finally associated with clinically significant drug resistance. As the disease progressed, the number of P-glycoprotein positive cells and the expression levels in individual cells increased.

### Circumvention of MDR by resistance modifying agents

Tsuruo *et al.* made the exiting observation that noncytotoxic doses of the calcium channel blocker verapamil could restore the sensitivity to Vinca alkaloids in MDR cells (Tsuruo *et al.*, 1981). As of now, a large number of such so-called resistance modifying agents (RMAs) has been found including: other calcium antagonists, e.g. diltiazem, nicardipine, niludipine (Tsuruo *et al.*, 1985); phenothiazines (Ford *et al.*, 1989); indole alkaloids, e.g. reserpine (Beck *et al.*, 1988) and reserpine analogs (Pearce *et al.*, 1989) as well as other alkaloids and amines (Zamora *et al.*, 1988); analogs of triparanol, e.g. tamoxifen (Ramu *et al.*, 1984), dipyridamole (Ramu & Ramu, 1989), and dihydropyridine (Nogae *et al.*, 1989) and cyclosporins (Nooter *et al.*, 1989; Slater *et al.*, 1986; Twentyman, 1988). For a number of these substances, structure/activity relationship studies have indicated physical and chemical features necessary to modulate MDR (Beck *et al.*, 1988; Ford *et al.*, 1989; Pearce *et al.*, 1989; Ramu & Ramu, 1989). In most cases, the reversal of resistance by RMAs is accompanied by increased accumulation of cytotoxic agents by the resistant cells as determined by radiolabelled drugs, fluorescence microscopy or laser flow cytometry (Hofsli & Nissen-Meyer, 1990; Kessel & Wilberding, 1985; Krishan *et al.*, 1986; Nooter *et al.*, 1989; Tsuruo *et al.*, 1984; Tsuruo *et al.*, 1982; Willingham *et al.*, 1986; Yalowich & Ross, 1985). The current hypothesis on the mode of action of RMAs is that they correct the defective cytotoxic drug accumulation by competing for outward transport directly through an interaction, i.e., binding with P-glycoprotein (Akiyama *et al.*, 1988; Cornwell *et al.*, 1987; Foxwell *et al.*, 1989; Naito & Tsuruo, 1989; Safa, 1988).

### Clinical trials with resistance modifying agents

The finding that elevated *mdr1* expression can occur in tumours and that specific agents can circumvent MDR in model systems has stimulated the development of clinical protocols in which RMAs are used in conjunction with cytotoxic drugs. Pilot studies and phase I/II trials using different RMAs and MDR related cytotoxic drugs in cancer patients have been reported. Verapamil was used with doxorubicin in ovarian cancer (Ozols *et al.*, 1987) and with vinblastine and VP-16 in pediatric drug resistant tumours (Cairo *et al.*, 1989). Verapamil was also used in combination with tamoxifen and doxorubicin, vincristine plus etoposide as the initial chemotherapy in small cell lung cancer (Figueredo *et al.*, 1990). The combination of trifluoperazine and doxorubicin was given for a variety of refractory malignancies (Miller *et al.*, 1988). In colon and renal cancer, cyclosporin A was combined with epidoxorubicin and vinblastine, respectively (Verweij *et al.*, 1990). Epidoxorubicin was also given in combination with quinidine as first line chemotherapy in advanced breast cancer (Jones *et al.*, 1990). These investigations, which were primarily intended as feasibility studies, have shown that there is no dramatic increased toxicity for normal tissues such as renal, hepatic, or intestinal epithelia with high levels of P-glycoprotein. There was also no evidence that RMAs potentiated the acute toxicities of the cytotoxic drugs. The clinical efficacy of the experimental protocols was assessed by the occurrence of otherwise unexpected tumour responses and the results overall are disappointing. A shortcoming of the above mentioned studies is a lack of data on P-glycoprotein expression in the tumours, making an evaluation difficult. More suitable for future studies would seem to be the haematological malignancies, because of the possibility of repeated tumour sampling.

Promising results have been obtained in two studies, one in multiple myeloma (Dalton *et al.*, 1989*a*) and another in acute myelocytic leukaemia (Sonneveld & Nooter, 1990). Verapamil was added to the standard regimen of vincristine, doxorubicin and dexamethasone (VAD) in patients with refractory multiple myeloma (7) or non-Hodgkin's lymphoma (1) (Dalton *et al.*, 1989*a*). Objective clinical responses were observed in three of eight patients who previously had been refractory to vincristine and doxorubicin. Six of these eight patients had evidence of P-glycoprotein expression in their tumour cells; of these, two showed a partial response and one gave a complete response for 6 months. However, three of six P-glycoprotein positive patients did not show objective response with the combined VAD + verapamil treatment.

From a therapeutic point of view, important features of MDR cells are their reduced drug accumulation and the resulting reduced drug sensitivity, which can both be restored by RMAs. We have shown that, in leukaemic cells expressing *mdr1*, the steady-state accumulation of daunorubicin could be significantly increased by cyclosporin A or verapamil (Herweijer *et al.*, 1990; Nooter *et al.*, 1990*b*). Since these RMAs inhibit the *mdr1* encoded drug pump, our data suggest that this pump is functional in leukaemias expressing the *mdr1* gene. We recently reported treatment of a refractory AML patient with daunorubicin and cytarabine combined with cyclosporin A (Sonneveld & Nooter, 1990). In that case, the emergence of the MDR phenotype was monitored during clinical progression of the disease. At relapse, a decrease in daunorubicin accumulation by AML blasts was associated with elevated *mdr1* expression and a decreased *in vitro* sensitivity to daunorubicin. Intracellular daunorubicin accumulation and *in vitro* sensitivity could be completely restored by adding cyclosporin A to the cells. During progressive relapse, the patient was treated with reinduction therapy to which cyclosporin A was added and this resulted in elimination of the *mdr1* positive AML clone. After 12 weeks, the resistant *mdr1* expressing clone reappeared in the blood and bone marrow.

In our opinion, future studies along this line in haematological malignancies should preferably include the following:

(a) RMAs are added to the cytotoxic protocols as early as possible in the development of clinical drug resistance; and,

(b) the efficacy of the currently used protocols, and those to which RMAs are added, in killing *mdr1* expressing tumour cells in relationship to the level of *mdr1* expression are monitored by *in situ* methods.

Since *mdr1* is also frequently expressed in untreated haematological malignancies, combination therapy should also be considered in previously untreated patients.

Another point of consideration is that the pharmacokinetics and, as a consequent of that the toxicity and efficacy of cytotoxic drugs, might be influenced by the simultaneous use of RMAs (Bright & Buss, 1990; Fedeli *et al.*, 1989; Kerr *et al.*, 1986; Nooter *et al.*, 1987). Therefore, we strongly recommend animal studies in which pharmacokinetics, optimal schedules and toxicology of combined drugs can be determined.

Supported by a grant from the Dutch Cancer Society. Dr J. Verweij is thanked for helpful discussions and critical reading of the manuscript.

### References

AKIYAMA, S., CORNWELL, M.M., KUWANO, M., PASTAN, I. & GOTTESMAN, M.M. (1988). Most drugs that reverse multidrug resistance also inhibit photoaffinity labeling of P-glycoprotein by a vinblastine analog. *Mol. Pharmacol.*, **33**, 144.

ARONOW, B., WATTS, T., LASSETTER, J., WASHTIEN, W. & ULLMAN, B. (1984). Biochemical phenotype of 5-fluorouracil-resistant murine T lymphoblasts with genetically altered CTP synthetase activity. *J. Biol. Chem.*, **259**, 9033.

BECH-HANSEN, N.T., TILL, J.E. & LING, V. (1976). Pleiotropic phenotype of colchicine-resistant CHO cells: cross-resistance and collateral sensitivity. *J. Cell Physiol.*, **88**, 23.

BECK, W.T., CIRTAIN, M.C., GLOVER, C.J., FELSTED, R.L. & SAFA, A.R. (1988). Effects of indole alkaloids on multidrugs resistance and labeling of P-glycoprotein by a photoaffinity analog of vinblastine. *Biochem. Biophys. Res. Comm.*, **153**, 959.

BEDFORD, P. & FOX, B.W. (1982). Repair of DNA interstrand crosslinks after busulphan. A possible mode of resistance. *Cancer Chemother. Pharmacol.*, **8**, 3.

BELL, D.R., GERLACH, J.H., KARTNER, N., BUICK, R.N. & LING, V. (1985). Detection of P-glycoprotein in ovarian cancer: a molecular marker associated with multidrug resistance. *J. Clin. Oncol.*, **3**, 311.

BOURHIS, J., BÉRNARD, J., HARTMANN, O., BOCCON-GIBOD, L., LEMERLE, J. & RIOU, G. (1989*a*). Correlation of MDR1 gene expression with chemotherapy in neuroblastoma. *J. Natl Cancer Inst.*, **81**, 1401.

BOURHIS, J., GOLDSTEIN, L.J., RIOU, G., PASTAN, I., GOTTESMAN, M.M. & BÉNARD, J. (1989*b*). Expression of a human multidrug resistance gene in ovarian carcinomas. *Cancer Res.*, **49**, 5062.

BRADLEY, G., JURANKA, P.F. & LING, V. (1988). Mechanism of multidrug resistance. *Biochim. Biophys. Acta.*, **948**, 87.

BRIGHT, J.M. & BUSS, D.D. (1990). Effects of verapamil on chronic doxorubicin-induced cardiotoxicity in dogs. *J Natl Cancer Inst.*, **82**, 963.

CABRAL, F., SOBEL, M.E. & GOTTESMAN, M.M. (1980). CHO mutants resistant to colchicine, colcemid or griseofuluin have an altered betatubulin. *Cell*, **20**, 29.

CAIRO, M.S., SIEGEL, S., ANAS, N. & SENDER, L. (1989). Clinical trial of continuous infusion verapamil, bolus vinblastine, and continuous infusion VP-16 in drug-resistant pediatric tumors. *Cancer Res.*, **49**, 1063.

CALLEN, D.F., BAKER, E., SIMMERS, R.N., SESHADRI, R. & RONINSON, I.B. (1987). Localization of the human multiple drug resistance gene, *MDR1* to 7q21.1. *Hum. Genet.*, **77**, 142.

CHAN, H.S.L., THORNER, P.S., HADDAD, G. & LING, V. (1990). Immunohistochemical detection of P-glycoprotein: prognostic correlation in soft tissue sarcoma of childhood. *J. Clin. Oncol.*, **8**, 689.

CHEN, C., CHIN, J.E., UEDA, K. & 4 others (1986). Internal duplication and homology with bacterial transport proteins in the mdr 1 (P-glycoprotein) gene from multidrug-resistant human cells. *Cell*, **47**, 381.

CHIN, J.E., SOFFIR, R., NOONAN, K.E, CHOI, K. & RONINSON, I.B. (1989). Structure and expression of the human MDR (P-glycoprotein) gene family. *Mol. Cell. Biol.*, **9**, 3808.

CHIN, K.V., TANAKA, S., DARLINGTON, G., PASTAN, I. & GOTTESMAN, M.M. (1990). Heat shock and arsenite increase expression of the multidrug resistance (MDR1) gene in human renal carcinoma cells. *J. Biol. Chem.*, **265**, 221.

CORDON-CARDO, C., O'BRIEN, J.P., BOCCIA, J., CASALS, D., BERTINO, J.R. & MELAMED, M.R. (1990). Expression of the multidrug resistance gene product (P-glycoprotein) in human normal and tumor tissues. *J. Histochem. Cytochem.*, **38**, 1277.

CORNWELL, M.M., PASTAN, I. & GOTTESMAN, M.M. (1987). Certain calcium channel blockers bind specifically to multidrug-resistant human KB carcinoma membrane vesicles and inhibit drug binding to P-glycoprotein. *J. Biol. Chem.*, **262**, 2166.

DALTON, W.S., GROGAN, T.M., MELTZER, P.S. & 5 others (1989*a*). Drug-resistance in multiple myeloma and non-Hodgkin's lymphoma: detection of P-glycoprotein and potential circumvention by addition of verapamil to chemotherapy. *J. Clin. Oncol.*, **7**, 415.

DALTON, W.S., GROGAN, T.M., RYBSKI, J.A. & 6 others (1989*b*). Immunohistochemical detection and quantitation of P-glycoprotein in multiple drug-resistant human myeloma cells: association with level of drug resistance and drug accumulation. *Blood*, **73**, 747.

DANØ, K. (1972). Cross-resistance between Vinca alkaloids and anthracyclines in Ehrlich ascites tumor in vivo. *Cancer Chemother. Rep.*, **56**, 701.

DANØ, K. (1973). Active outward transport of daunomycine in resistant Ehrlich ascites tumor cells. *Biochim. Biophys. Acta.*, **323**, 466.

EPSTEIN, J., XIAO, H. & OBA, B.K. (1989). P-glycoprotein expression in plasma-cell myeloma is associated with resistance to VAD. *Blood*, **74**, 913.

FEDELI, L., COLOZZA, M., BOSCHETTI, E. & 8 others (1989). Pharmacokinetics of vincristine in cancer patients treated with nifedipine. *Cancer*, **64**, 1805.

FIGUEREDO, A., ARNOLD, A., GOODYEAR, M. & 4 others (1990). Addition of verapamil and tamoxifen to the initial chemotherapy of small cell lung cancer. A phase I/II study. *Cancer*, **65**, 1895.

FLINTOFF, W.F. & ESSANI, K. (1980). Methotrexate resistance Chinese hamster ovary cells contain a dihydrofolate reductase with an altered affinity for methotrexate. *Biochemistry*, **19**, 4321.

FOJO, A.T., SHEN, D., MICKLEY, L.A., PASTAN, I. & GOTTESMAN, M.M. (1987*a*). Intrinsic drug resistance in human kidney cancer is associated with expression of a human multidrug-resistance gene. *J. Clin. Oncol.*, **5**, 1922.

FOJO, A.T., UEDA, K., SLAMON, D.J., POPLACK, D.G. GOTTESMAN, M.M. & PASTAN, I. (1987*b*). Expression of a multidrug-resistance gene in human tumors and tissues. *Proc. Natl Acad. Sci. USA*, **84**, 265.

FORD, J.M., PROZIALECK, W.C. & HAIT, W.N. (1989). Structural features determining activity of phenothiazines and related drugs for inhibition of cell growth and reversal of multidrug resistance. *Mol. Pharmacol.*, **35**, 105.

FOXWELL, B.M.J., MACKIE, A., LING, V. & RYFFEL, B. (1989). Identification of the multidrug resistance-related P-glycoprotein as a cyclosporine binding protein. *Mol. Pharmacol.*, **36**, 543.

GALSKI, H., SULLIVAN, M., WILLINGHAM, M.C. & 4 others (1989). Expression of a human multidrug resistance cDNA (MDR1) in the bone marrow of transgenic mice: resistance to daunomycin-induced leukopenia. *Mol. Cell. Biol.*, **9**, 4357.

GERLACH, J.H., BELL, D.R., KARAKOUSIS, C. & 5 others (1987). P-glycoprotein in human sarcoma: evidence for multidrug resistance. *J. Clin. Oncol.*, **5**, 1452.

GERLACH, J.H., ENDICOTT, J.A., JURANKA, P.F. & 4 others (1986). Homology between P-glycoprotein and a bacterial haemolysin transport protein suggests a model for multidrug-resistance. *Nature*, **324**, 485.

GOLDSTEIN, L.J., FOJO, A.T., UEDA, K. & 5 others (1990). Expression of the multidrug resistance, *MDR1*, gene in neuroblastomas. *J. Clin. Oncol.*, **8**, 128.

GOLDSTEIN, L.J., GALSKI, H., FOJO, A.T. & 11 others (1989). Expression of a multidrug resistance gene in human cancers. *J. Natl Cancer Inst.*, **81**, 116.

GROS, P., CROOP, J. & HOUSMAN, D. (1986). Mammalian multidrug resistance gene: complete cDNA sequence indicates strong homology to bacterial transport proteins. *Cell*, **47**, 371.

GROS, P., RAYMOND, M., BELL, J. & HOUSMAN, D. (1988). Cloning and characterization of a second member of the mouse *mdr* gene family. *Mol. Cell. Biol.*, **8**, 2770.

HAMADA, H. & TSURUO, T. (1986). Functional role for the 170- to 180-kDa glycoprotein specific to drug-resistant tumor cells as revealed by monoclonal antibodies. *Proc. Natl Acad. Sci. USA*, **83**, 7785.

HAMADA, H. & TSURUO, T. (1988). Characterization of the ATPase activity of the Mr 170,000 to 180,000 membrane glycoprotein (P-glycoprotein) associated with multidrug resistance in K562/ADM cells. *Cancer Res.*, **48**, 4926.

HERWEIJER, H., SONNEVELD, P., BAAS, F. & NOOTER, K. (1990). Expression of *mdr1* and *mdr3* multidrug-resistance genes in human acute and chronic leukemias and association with stimulation of drug accumulation by cyclosporine. *J. Natl Cancer Inst.*, **82**, 1133.

HOFSLI, E. & NISSEN-MEYER, J. (1990). Reversal of multidrug resistance by lipophilic drugs. *Cancer Res.*, **50**, 3997.

HOLMES, J., JACOBS, A., CARTER, G., JANOWSKA-WIECZOREK, A. & PADUA, R.A. (1989). Multidrug resistance in haemopoietic cell lines, myelodysplastic syndromes and acute myeloblastic leukaemia. *Br. J. Haematol.*, **72**, 40.

HOLMES, J.A., JACOBS, A., CARTER, G., WHITTAKER, J.A., BENTLEY, D.P. & PADUA, R.A. (1990). Is the mdr1 gene relevant in chronic lymphocytic leukemia? *Leukemia*, **4**, 216.

HORIO, M., GOTTESMAN, M.M. & PASTAN, I. (1988). ATP-dependent transport of vinblastine in vesicles from human multidrug-resistant cells. *Proc. Natl Acad. Sci. USA*, **85**, 3580.

INABA, M. & JOHNSON, R.K. (1977). Decreased retention of actinomycin D as the basis for cross-resistance in anthracycline-resistant sublines of P388 leukemia. *Cancer Res.*, **37**, 4629.

JONES, R.D., KERR, D.J., HARNETT, A.N., RANKIN, E.M., RAY, S. & KAYE, S.B. (1990). A pilot study of quinidine and epirubicin in the treatment of advanced breast cancer. *Br. J. Cancer*, **62**, 133.

KAKEHI, Y., KANAMARU, H., YOSHIDA, O. & 4 others (1988). Measurement of multidrug-resistance messenger RNA in urogenital cancers; elevated expression in renal cell carcinoma is associated with intrinsic drug resistance. *J. Urol.*, **139**, 862.

KANAMARU, H., KAKEHI, Y., YOSHIDA, O., NAKANISHI, S., PASTAN, I. & GOTTESMAN, M.M. (1989). MDR1 RNA levels in human renal cell carcinomas: correlation with grade and prediction of reversal of doxorubicin resistance by quinidine in tumor explants. *J. Natl Cancer Inst.*, **81**, 844.

KARTNER, N., EVERNDEN-PORELLE, D., BRADLEY, G. & LING, V. (1985). Detection of P-glycoprotein in multidrug-resistant cell lines by monoclonal antibodies. *Nature*, **316**, 820.

KEITH, W.N., STALLARD, S. & BROWN, R. (1990). Expression of *mdr1* and *gst-π* in human breast tumours: comparison to *in vitro* chemosensitivity. *Br. J. Cancer*, **61**, 712.

KERR, D.J., GRAHAM, J., CUMMINGS, J. & 4 others (1986). The effect of verapamil on the pharmacokinetics of adriamycin. *Cancer Chemother. Pharmacol.*, **18**, 239.

KESSEL, D. & BOSMANN, H.B. (1970). On the characteristics of actinomycin D resistance in L5178Y cells. *Cancer Res.*, **30**, 2695.

KESSEL, D. & WILBERDING, C. (1985). Anthracycline resistance in P388 murine leukemia and its circumvention by calcium antagonists. *Cancer Res.*, **45**, 1687.

KOHNO, K., SATO, S., TAKANO, H., MATSUO, K. & KUWANO, M. (1989). The direct activation of human multidrug resistance gene (mdr1) by anticancer agents. *Biochem. Biophys. Res. Commun.*, **165**, 1415.

KRISHAN, A., SAUERTEIG, A., GORDON, K. & SWINKIN, C. (1986). Flow cytometric monitoring of cellular anthracycline accumulation in murine leukemic cells. *Cancer Res.*, **46**, 1768.

LAI, S., GOLDSTEIN, L.J., GOTTESMAN, M.M. & 7 others (1989). MDR1 gene expression in lung cancer. *J. Natl Cancer Inst.*, **81**, 1144.

MA, D.D.F., DAVEY, R.A., HARMAN, D.H. & 5 others (1987). Detection of a multidrug resistant phenotype in acute non-lymphoblastic leukaemia. *Lancet*, **i**, 135.

MILLER, R.L., BUKOWSKI, R.M., BUDD, G.T. & 5 others (1988). Clinical modulation of doxorubicin resistance by the calmodulin-inhibitor, trifluoperazine: a phase I/III trial. *J. Clin. Oncol.*, **6**, 880.

MOSCOW, J.A. & COWAN, K.H. (1990). Multidrug resistance. In *Cancer Chemotherapy and Biological Response Modifiers Annual 11*, Pinedo, H.M., Chabner, B.A. & Longo, D.L. (eds) p. 97. Elsevier Science Publishers: Amsterdam.

MOSCOW, J.A., FAIRCHILD, C.R., MADDEN, M.J. & 7 others (1989). Expression of anionic glutathione-S-transferase and P-glycoprotein genes in human tissues and tumors. *Cancer Res.*, **49**, 1422.

NAITO, M. & TSURUO, T. (1989). Competitive inhibition by verapamil of ATP-dependent high affinity vincristine binding to the plasma membrane of multidrug-resistant K562 cells without calcium ion involvement. *Cancer Res.*, **49**, 1452.

NG, W.F., SARANGI, F., ZASTAWNY, R.L., VEINOT-DREBOT, L. & LING, V. (1989). Identification of members of the P-glycoprotein multigene family. *Mol. Cell. Biol.*, **9**, 1224.

NOGAE, I., KOHNO, K., KIKUCHI, J. & 8 others (1989). Analysis of structural features of dihydropyridine analogs needed to reverse multidrug resistance and to inhibit photoaffinity labeling of P-glycoprotein. *Biochem. Pharmacol.*, **38**, 519.

NOOTER, K., OOSTRUM, R. & DEURLOO, J. (1987). Effects of verapamil on the pharmacokinetics of daunomycin in the rat. *Cancer Chemother. Pharmacol.*, **20**, 176.

NOOTER, K., OOSTRUM, R., JONKER, R. VAN DEKKEN, H., STOKDIJK, W. & VAN DEN ENGH, G. (1989). Effect of cyclosporin A on daunorubicin accumulation in multidrug-resistant P388 leukemia cells measured by real-time flow cytometry. *Cancer Chemother. Pharmacol.*, **23**, 296.

NOOTER, K., SONNEVELD, P., JANSSEN, A. & 6 others (1990*a*). Expression of the *mdr3* gene in prolymphocytic leukemia: association with cyclosporin-A-induced increase in drug-accumulation. *Int. J. Cancer*, **45**, 626.

NOOTER, K., SONNEVELD, P., OOSTRUM, R., HERWEIJER, H., HAGENBEEK, A. & VALERIO, D. (1990*b*). Overexpression of the *mdr1* gene in blast cells from patients with acute myelocytic leukemia is associated with decreased anthracycline accumulation that can be restored by cyclosporin-A. *Int. J. Cancer*, **45**, 263.

OZOLS, R.F., CUNNION, R.E., KLECKER, R.W. & 4 others (1987). Verapamil and adriamycin in the treatment of drug-resistant ovarian cancer patients. *J. Clin. Oncol.*, **5**, 641.

PEARCE, H.L., SAFA, A.R., BACH, N.J., WINTER, M.A., CIRTAIN, M.C. & BECK, W.T. (1989). Essential features of the P-glycoprotein pharmacophore as defined by a series of reserpine analogs that modulate multidrug resistance. *Proc. Natl Acad. Sci. USA*, **86**, 5128.

PIRKER, R., GOLDSTEIN, L.J., LUDWIG, H. & 4 others (1989). Expression of a multidrug resistance gene in blast crisis of chronic myelogenous leukemia. *Cancer Commun.*, **1**, 141.

RAMU, A., GLAUBIGER, D. & FUKS, Z. (1984). Reversal of acquired resistance to doxorubicin in P388 murine leukemia cells by tamoxifen and other triparanol analogues. *Cancer Res.*, **44**, 4392.

RAMU, N. & RAMU, A. (1989). Circumvention of adriamycin resistance by dipyridamole analogues: a structure-activity relationship study. *Int. J. Cancer*, **43**, 487.

REDWOOD, W.R. & COLVIN, M. (1980). Transport of melphalan by sensitive and resistant L1210 cells. *Cancer Res.*, **40**, 1144.

RIEHM, H. & BIEDLER, J.L. (1972). Potentiation of drug effect by Tween 80 in Chinese hamster cells resistant to actinomycin D and daunomycin. *Cancer Res.*, **32**, 1195.

RONINSON, I.B., CHIN, J.E., CHOI, K. & 6 others (1986). Isolation of human mdr DNA sequences amplified in multidrug-resistant KB carcinomas cells. *Proc. Natl Acad. Sci. USA*, **83**, 4538.

ROTHENBERG, M.L., MICKLEY, L.A., COLE, D.E. & 4 others (1989). Expression of the *mdr1*/P-170 gene in patients with acute lymphoblastic leukemia. *Blood*, **74**, 1388.

SAFA, A.R. (1988). Photoaffinity labeling of the multidrug-resistance-related P-glycoprotein with photoactive analogs of verapamil. *Proc. Natl Acad. Sci. USA*, **85**, 7187.

SALMON, S.E., GROGAN, T.M., MILLER, T., SCHEPER, R. & DALTON, W.S. (1989). Prediction of doxorubicin resistance *in vitro* in myeloma, lymphoma, and breast cancer by P-glycoprotein staining. *J. Natl Cancer Inst.*, **81**, 696.

SATO, H., GOTTESMAN, M.M., GOLDSTEIN, L.J. & 4 others (1990). Expression of the multidrug resistance gene in myeloid leukemias. *Leukemia Res.*, **14**, 11.

SCHNEIDER, J., BAK, M., EFFERTH, T., KAUFMANN, M., MATTERN, J. & VOLM, M. (1989). P-glycoprotein expression in treated and untreated human breast cancer. *Br. J. Cancer*, **60**, 815.

SIROTNAK, F.M., MOCCIO, D.M., KELLEHER, L.E. & GOUTAS, L.J. (1981). Relative frequency and kinetic properties of transport-defective phenotypes among methotrexate-resistant L1210 clonal cell lines derived *in vivo*. *Cancer Res.*, **41**, 4447.

SIROTNAK, F.M., YANG, C., MINES, L.S., ORIBÉ, E. & BIEDLER, J.L. (1986). Markedly altered membrane transport and intracellular binding of vincristine in multidrug-resistant Chinese hamster cells selected in resistance to vinca alkaloids. *J. Cell. Physiol.*, **126**, 266.

SKOVSGAARD, T. (1978). Mechanism of cross-resistance between vincristine and daunorubicin in Ehrlich ascites tumor cells. *Cancer Res.*, **38**, 4722.

SLATER, L.M., SWEET, P., STUPECKY, M. & GUPTA, S. (1986). Cyclosporin A reverses vincristine and daunorubicin resistance in acute lymphatic leukemia *in vitro*. *J. Clin. Invest.*, **77**, 1405.

SONNEVELD, P. & NOOTER, K. (1990). Reversal of drug-resistance by cyclosporin-A in a patient with acute myelocytic leukaemia. *Br. J. Haematol.*, **75**, 208.

THIEBAUT, F., TSURUO, T., HAMADA, H., GOTTESMAN, M.M., PASTAN, I. & WILLINGHAM, M.C. (1987). Cellular localization of the multidrug-resistance gene product P-glycoprotein in normal human tissues. *Proc. Natl Acad. Sci. USA*, **84**, 7735.

TSURUO, T., IIDA, H., KITATANI, Y., YOKOTA, K., TSUKAGOSHI, S. & SAKURAI, Y. (1984). Effects of quinidine and related compounds on cytotoxicity and cellular accumulation of vincristine and adriamycin in drug-resistant tumor cells. *Cancer Res.*, **44**, 4303.

TSURUO, T., IIDA, H., TSUKAGOSHI, S. & SAKURAI, Y. (1981). Overcoming of vincristine resistance in P388 leukemia *in vivo* and *in vitro* through enhanced cytotoxicity of vincristine and vinblastine by verapamil. *Cancer Res.*, **41**, 1967.

TSURUO, T., IIDA, H., TSUKAGOHSI, S. & SAKURAI, Y. (1982). Increased accumulation of vincristine and adriamycin in drug-resistant P388 tumor cells following incubation with calcium antagonists and calmodulin inhibitors. *Cancer Res.*, **42**, 4730.

TSURUO, T., KAWABATA, H., NAGUMO, N. & 4 others (1985). Potentiation of antitumour agents by calcium channel blockers with special references to cross-resistance patterns. *Cancer Chemother. Pharmacol.*, **15**, 16.

TSURUO, T., SUGIMOTO, Y., HAMADA, H. & 5 others (1987). Detection of multidrug resistance markers, P-glycoprotein and *mdr*1 mRNA, in human leukemia cells. *Jpn. J. Cancer Res. (Gann)*, **78**, 1415.

TWENTYMAN, P.R. (1988). Modification of cytotoxic drug resistance by non-immunosuppressive cyclosporins. *Br. J. Cancer*, **57**, 254.

UEDA, K., CARDARELLI, C., GOTTESMAN, M.M. & PASTAN, I. (1987). Expression of a full-length cDNA for the human '*MDR1*' gene confers resistance to colchicine, doxorubicin, and vinblastine. *Proc. Natl Acad. Sci. USA*, **84**, 3004.

VAN DER BLIEK, A.M., BAAS, F., TEN HOUTE-DE LANGE, T., KOOIMAN, P.M., VAN DER VELDE-KOERTS, T. & BORST, P. (1987). The human *mdr3* gene encodes a novel P-glycoprotein homologue and gives rise to alternatively spliced mRNAs in liver. *EMBO J.*, **6**, 3325.

VAN DER BLIEK, A.M., BAAS, F., VAN DER VELDE-KOERTS, T. & 6 others (1988*b*). Genes amplified and overexpressed in human multidrug-resistant cell lines. *Cancer Res.*, **48**, 5927.

VAN DER BLIEK, A.M. & BORST, P. (1989). Multidrug-Resistance. In *Advances in Cancer Research 52*, van de Woude, G.F. & Klein, G. (eds) p. 165. Academic Press: New York.

VAN DER BLIEK, A.M., KOOIMAN, P.M., SCHNEIDER, C. & BORST, P. (1988*a*). Sequence of *mdr3* cDNA encoding a human P-glycoprotein. *Gene*, **71**, 401.

VAN DER VALK, P., VAN KALKEN, C.K., KETELAARS, H. & 8 others (1990). Distribution of multidrug resistance-associated P-glycoprotein in normal and neoplastic human tissues. *Ann. Oncol.*, **1**, 56.

VERWEIJ, J., HERWEIJER, H., PLANTING, A.S.T. & 4 others (1990). *In vitro* and *in vivo* studies on the effect of cyclosporin-A (Cy-A) in the circumvention of clinical multidrug resistance (MDR). *Proc. Am. Soc. Clin. Oncol.*, **9**, 74.

WEINSTEIN, R.S., KUSZAK, J.R., KLUSKENS, L.F. & COON, J.S. (1990). P-glycoproteins in pathology – the multidrug resistance gene family in humans. *Hum. Pathol.*, **21**, 34.

WILLINGHAM, M.C., CORNWELL, M.M., CARDARELLI, C.O., GOTTESMAN, M.M. & PASTAN, I. (1986). Single cell analysis of daunomycin uptake and efflux in multidrug-resistant and -sensitive KB cells: effect of verapamil and other drugs. *Cancer Res.*, **46**, 5941.

YALOWICH, J.C. & ROSS, W.E. (1985). Verapamil-induced augmentation of etoposide accumulation in L1210 cells *in vitro*. *Cancer Res.*, **45**, 1651.

ZAMORA, J.M., PEARCE, H.L. & BECK, W.T. (1988). Physical-chemical properties shared by compounds that modulate multidrug resistance in human leukemic cells. *Mol. Pharmacol.*, **33**, 454.